# Immune Modulation in Primary *Vaccinia virus* Zoonotic Human Infections

**DOI:** 10.1155/2012/974067

**Published:** 2011-12-20

**Authors:** Juliana Assis Silva Gomes, Fernanda Fortes de Araújo, Giliane de Souza Trindade, Bárbara Resende Quinan, Betânia Paiva Drumond, Jaqueline Maria Siqueira Ferreira, Bruno Eduardo Fernandes Mota, Maurício Lacerda Nogueira, Erna Geessien Kroon, Jônatas Santos Abrahão, Rodrigo Côrrea-Oliveira, Flávio Guimarães da Fonseca

**Affiliations:** ^1^Departamento de Morfologia, Instituto de Ciências Biológicas, Universidade Federal de Minas Gerais (UFMG), Avenida Antônio Carlos 6627, 31270-901 Belo Horizonte, MG, Brazil; ^2^Instituto René Rachou (IRR), Fundação Oswaldo Cruz, Avenida Augusto de Lima 1715, 30190-002 Belo Horizonte, MG, Brazil; ^3^Departamento de Microbiologia, Instituto de Ciências Biológicas, Universidade Federal de Minas Gerais (UFMG), Avenida Antônio Carlos 6627, 31270-901 Belo Horizonte, MG, Brazil; ^4^Departamento de Doenças Infecciosas e Parasitárias, Faculdade de Medicina de São José do Rio Preto (FAMERP), Avendia Brigadeiro Faria Lima 5416, 15090-000 São José do Rio Preto, SP, Brazil

## Abstract

In 2010, the WHO celebrated the 30th anniversary of the smallpox eradication. Ironically, infections caused by viruses related to smallpox are being increasingly reported worldwide, including *Monkeypox*, *Cowpox,* and *Vaccinia virus* (VACV). Little is known about the human immunological responses elicited during acute infections caused by orthopoxviruses. We have followed VACV zoonotic outbreaks taking place in Brazil and analyzed cellular immune responses in patients acutely infected by VACV. Results indicated that these patients show a biased immune modulation when compared to noninfected controls. Amounts of B cells are low and less activated in infected patients. Although present, T CD4^+^ cells are also less activated when compared to noninfected individuals, and so are monocytes/macrophages. Similar results were obtained when Balb/C mice were experimentally infected with a VACV sample isolated during the zoonotic outbreaks. Taking together, the data suggest that zoonotic VACVs modulate specific immune cell compartments during an acute infection in humans.

## 1. Introduction

Thirty years after smallpox eradication the interest in *Orthopoxvirus *infections has been renewed by the potential use of Smallpox as a biological weapon [1] and the substantial increase in reports of zoonotic poxvirus infections throughout the world [2, 3], including the emergence of *Monkeypox virus *(MPV) in Africa and the USA [4], the emergence of *Vaccinia virus *(VACV) infections in Brazil [5–10], the maintenance of VACV in milking buffaloes in India [11, 12], and the increasing numbers of *Cowpox virus *(CPV) infections in Europe and Central Asia [13].

At the time of smallpox eradication, human immune responses to the *Variola virus *infection were not well understood, nor the response against live VACV strains used for vaccination. In the last years, however, our knowledge on how humans respond immunologically to *Orthopoxvirus *infections was greatly improved [14–20]. Most efforts have been directed to understand the mechanisms of protection against subsequent infections conferred by previous vaccination. In this respect, it is now clear that antibodies have a major role in long-term protection against Orthopoxviruses, with relatively high titers that remain stable for decades, whereas CD8^+^ and CD4^+^ T cell responses decline slowly over time [14, 21–23]. On the other hand, the immune mechanisms involved in replication control and clearing of an *Orthopoxvirus *primary infection are still not fully understood, but essential roles for the innate and adaptive immunity have been demonstrated [24]. In the innate immunity context, studies have shown that the complement system and NK cells are important to control these infections until the adaptive responses arise; the loss of these functions results frequently in host death [25–27]. For the adaptive responses, the importance and efficiency of CD8^+^ T cells to control and clear poxviruses in the absence of antibody production depend greatly on the animal model and the virus species used [20]. Studies using VACV inoculation in mice have shown that CD4^+^ T and B cells are able and sufficient to eliminate the virus. However, this is apparently not the case when animals are infected with species-specific orthopoxviruses such as *Ectromelia virus *[28–30]. In the latter case, CD8^+^ T cells are vital to contain the virus early in infection, as mice lacking CD8^+^ T cells succumb early due to high viral load. However, CTL function alone is insufficient to clear the virus. At later stages, antibodies become essential for virus elimination and survival. Evidences come from studies showing the inability of animals deficient in B or CD4^+^ T cells to effectively control and clear the infection [29–31]. Moreover, passive transfer of B cells or immune serum restores virus elimination capabilities in infected animals [30]. The requirement for CD4^+^ T cells is clear as robust-specific antibody responses fail to develop in animals lacking these cells [28]. Similarly, CD4^+^ T cell function is essential for an optimal CTL response [20]. The most likely scenario is that both cell and humoral immunities work complementarily to contain *Orthopoxvirus *acute infections.

The active circulation of orthopoxviruses in Brazil has been reported since the early 1960s. From 1999 onwards, many outbreaks of an exanthematic disease affecting humans and cattle alike were associated with such viruses. As isolates became available, the agent of such outbreaks was demonstrated to be the VACV. Infections are usually zoonotic, as the virus spreads from sick lactating cows to their handlers, leading to the formation of vesicle and ulcers on the hands, arms, torso and face of sick individuals (reviewed in [32, 33]).

Here we analyzed aspects of the cellular immune responses in patients acutely and naturally infected by VACV during zoonotic outbreaks taking place in Brazil. Our results indicate that these infections trigger a virus-specific immune modulation biased mainly towards macrophage and T CD4^+^ and B cell functions.

## 2. Materials and Methods

### 2.1. Study Population

The study population consisted of 53 individuals showing signs of poxvirus infection, ages between 18 and 70 years, both genders, all living in the outbreak areas. Patients were classified as acutely infected on the basis of the occurrence of typical clinical symptoms (mainly the presence of nonhealed pustules and vesicles), VACV DNA detection in serum samples or lesion fluids, and virus isolation from lesion swabs. Eighteen healthy individuals, with no signs of infection, 29 to 55 years old, also residing at the outbreak areas, were enlisted and included in the study as a noninfected control group. All patients were properly examined by a physician and those presenting apparent clinical signs of any other disease, infectious or not, were not included in the study.

### 2.2. Virus Isolation from Animals and Humans

Fluid from suppurated lesions was collected using a sterile swab and maintained in MEM culture media for transportation. Viruses were isolated by inoculation in chorio-allantoic membranes of embryonated chicken eggs (CAMs) and amplified in VERO cells. Viruses were purified and characterized by neutralization tests using anti-VACV antibodies and by nucleotide sequencing of *Orthopoxvirus-*specific genes after PCR amplification using VACV-specific primers [34].

### 2.3. Phylogenetic Analysis

The hemagglutinin (HA) gene nucleotide sequences from the isolated viruses and from other Orthopoxviruses (retrieved from GenBank) were aligned on the basis of codon positions using the CLUSTAL W software. Alignments were manually edited and used to perform phylogenetic analyses using the Neighbor-joining and Maximum-likelihood methods implemented in Mega3 and Paup*4.0b10. GenBank accession numbers are as follows: Vaccinia virus: Western Reserve (VACV-WR) (AY243312), VACV-Lister (AY678276), Modified virus Ankara (VACV-MVA) (AY603355), Copenhagen (VACV-COP) (M35027), VACV-Wyeth (Z99051), VACV-TTan (U25662), VACV-Malbran (AY146624), Br-Hu-1 (FJ173000), Br-Hu-2 (EF063677), Br-An-1 (FJ173001), Br-An-2 (FJ173002), Br-An-3 (FJ173003), Passatempo (VACV-PSTV) (DQ070848), Cantagalo (VACV-CTGV) (AF229247), Araçatuba (VACV-ARAV) (AY523994), Guarani P2 (VACV-GP2V) (DQ206437), Muriaé (VACV-MURV) (DQ247770), VACV-BeAn58058 (DQ206442), Belo Horizonte (VACV-VBH) (DQ206435), VACV-IOC (AF229248), Guarani P1 (VACV-GP1V) (DQ206436), VACV-SPAn232 (DQ222922), Lister Butantan (VACV-LTBUT) (EF175985); Buffalopox virus (BFPV-3906) (AF375077); Cowpox virus Brighton Red (CPXV-BR) (AF482758)'Rabbitpox virus rev (RBPV-rev) (AY484669); Ectromelia virus Moscow (ECTV) (AF012825); Camelpox virus: CMS (CMLV-CMS) (AY009089), M-96 (CMLV-M96) (AF438165); Variola virus: Garcia-1966 (VARV-GAR) (U18338) and Bangladesh-1975 (VARV-BSH) (L22579).

### 2.4. Cell Preparation and Proliferation Assay

Peripheral blood mononuclear cells (PBMCs) were isolated by Ficoll-diatriazoate density gradient centrifugation (LSM, Organon Teknica, Charleston, SC), cultured in triplicate (10^6^ cells/well) in 96-well flat-bottom plates, and stimulated with either UV-inactivated VACV-WR for 6 days or PHA (at 2.5 *μ*g/mL to test cell viability) for 3 days. Tritiated thymidine (1 *μ*Ci/well) was added to the cultures for 6 hours before completion of the incubation period. Incorporation of [^3^H]thymidine was determined by liquid scintillation counting. Data were analyzed and presented as countings per minute (CPM) (calculated as the mean experimental cpm ± SD − mean control cpm ± SD).

### 2.5. Detection of Cytokine Levels by Cytometric Bead Array Immunoassay (CBA)

Microbeads consisted of six distinct populations, unique on their Type 3 fluorescence intensity (FL-3), each coupled to mAb against one of the six Th1/Th2 and regulatory cytokines (IL-2, IL-4, IL-5, IL-10, TNF-*α*, and IFN-*γ*). Captured cytokines were detected using six different mAbs coupled to PE (FL-2). A total of 1,800 events/gate were acquired. Standard curves were plotted using a four-parameter logistic curve fitting model. Cytokine concentrations were determined using standard curves. If a sample had a cytokine concentration below the detection limit for the assay, a value of 0 was attributed for statistical purposes.

### 2.6. Cell Phenotype Analysis

Cells were quantified after *in vitro *antigenic stimulation with UV-inactivated VACV using mouse anti-human monoclonal antibodies (MoAbs) conjugated with FITC or PE, specific for cell-surface markers. Cultured cells were washed in PBS containing 1% BSA plus 0.1% sodium azide (FACS buffer) and stained with monoclonal antibodies against CD3, CD4, and CD8 for T cell populations, CD19 for B cells, CD16 and CD56 for NK cells, and CD14 for monocytes. Same cells were labeled simultaneously with antibodies against costimulatory molecules (HLA-DR, CD25, CD69, CD28, CD80, and CD86). Cell preparations were fixed in FACS fix solution and stored at 4°C in the dark. A total of 30,000 events/tube were acquired using a FACScalibur flow cytometer (Becton Dickinson) set up to measure forward (FSC), side (SSC) light scatters, FITC (FL-1), and PE (FL-2) fluorescence. CELLQuest software was used for data acquisition and analysis.

### 2.7. Statistical Analysis

Analyses were performed using GraphPad Prism version 3.0 software. The following nonparametric tests were performed: (1) Mann-Whitney test to compare two groups (noninfected × infected individuals); and (2) Wilcoxon test to compare cultures stimulated and nonstimulated. The statistical analysis was performed by using the median values of each group.

### 2.8. Animal Experiments

Groups of 10 four-week-old male Balb/C mice were used. Animals were intranasally infected with 10^4^ PFUs of a zoonotic VACV sample in PBS. Ten days after infection, animals were anesthetized and blood was collected. PBMCs were obtained as mentioned and cell surface markers (CD4, CD14, CD25, and CD69) were detected as described above.

### 2.9. Ethics Statement

The human study protocol complied with the Brazilian National Council of Health regulations and was approved by the Instituto René Rachou Review Board (IRR IRB) under protocol number 03/2006. All patients signed informed consents. Animal experiments were conducted in accordance with the Brazilian Federal Law number 11.794 (October 8th, 2008), which regulates the scientific use of animals, and IACUC guidelines. All protocols were approved by the Committee of Ethics for Animal Experimentation (CETEA) at UFMG under permit 9/2009, valid through April 2014. The CETEA-UFMG is affiliated to the National Council of Animal Experimentation Control (CONCEA).

## 3. Results

### 3.1. Characterization of the Population Involved in the Study

Since 1999, yearly outbreaks of an exanthematic disease affecting humans and cattle have been reported among poor pockets of population in the rural countryside of Southeast Brazil. In most cases, the isolated infectious agent causing the zoonotic outbreaks was the VACV. We have followed outbreaks taking place in farms at the Minas Gerais State, SE, Brazil, from 2005 to 2009. The studied group consisted of 53 affected human patients, who were clinically evaluated, and 18 noninfected and healthy individuals living at the same areas affected by the outbreaks (control group). Clinical symptoms of the infection included high fever, headache, muscle pain, nausea, lymphangitis, and the appearance of pleiomorphic lesions on hands, forelimbs and eventually in the face, torso, and genitals. In all patients, acute lesions were associated with a roseolar erythema and localized edema leading to the formation of vesicles [8]. As pointed out in previous studies [5–8], the disease is occupational, as persons dealing with infected dairy cattle were those presenting signs of infection. Importantly, out of 53 infected patients, at least 10 were vaccinated against smallpox in the past, as confirmed by visualization of a typical vaccination scar in their left arm.

### 3.2. Virus Samples Isolated during the Studied Outbreaks Are Genetically Consistent with Previously Described Circulating VACV Isolates

**Figure 1:**
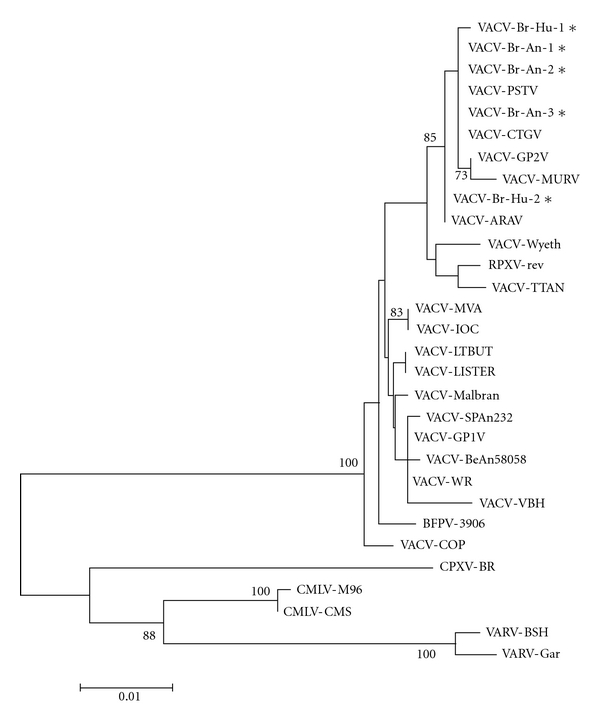
Phylogenetic relationships between the VACV samples obtained in this study and other relevant poxviruses. The phylogenetic tree was constructed by the Neighbor-joining method and used hemagglutinin gene nucleotide sequences from various orthopoxviruses, including Brazilian *Vaccinia virus *(VACV) isolates and other poxviruses. The Tamura3-parameter nucleotide substitution model was used and the reliability of the branching patterns was tested by 1000 bootstrap pseudo-replicates. Bootstrap values above 70% are shown. The scale bar represents 1% nucleotide sequence divergence. Samples are as follows: Zoonotic Brazilian Vaccinia virus isolated from humans (VACV-Br-Hu-1, VACV-Br-Hu-2) or cattle (VACV-Br-An-1, VACV-Br-An-2, VACV-Br-An-3)—labeled with stars (∗); other *Vaccinia virus *strains isolated in Brazil—Passatempo (VACV-PSTV), Cantagalo (VACV-CTGV), Araçatuba (VACV-ARAV), Guarani P2 (VACV-GP2V) (DQ206437), Muriaé (VACV-MURV), VACV-BeAn58058, Belo Horizonte (VACV-VBH), VACV-SPAn232, Guarani P1 (VACV-GP1V); reference *Vaccinia virus *strains—Western Reserve (VACV-WR), VACV-Lister, Modified virus Ankara (VACV-MVA), Copenhagen (VACV-COP), VACV-Wyeth, VACV-TTan, VACV-Malbran, Lister Butantan (VACV-LTBUT); VACV-IOC (AF229248), Buffalopox virus (BFPV-3906) (AF375077); other Orthopoxviruses—Cowpox virus Brighton Red (CPXV-BR), *Rabbitpox virus *rev (RBPV-rev), Ectromelia virus Moscow (ECTV), *Camelpox virus *CMS (CMLV-CMS), *Camelpox virus *M-96 (CMLV-M96), *Variola virus *Garcia-1966 (VARV-GAR), and *Variola virus *Bangladesh-1975 (VARV-BSH).

Two virus isolates were obtained from human patients during the studied outbreaks and characterized, together with three other samples isolated from cattle at the same areas (herein referred as VACV-Br-Hu-1, VACV-Br-Hu-2, VACV-Br-An-1, VACV-Br-An-2, and VACV-Br-An-3). The hemagglutinin (HA) genes from all viruses were sequenced, and they presented a signature of 18-nucleotide deletion also observed in previously isolated Brazilian VACVs [5–7, 32, 34]. Phylogenetic analyses based on the HA nucleotide sequences demonstrated that all isolated viruses cluster together with other Brazilian VACV samples isolated in the past, and none cluster with attenuated vaccine strains including VACV-Lister, the Lister-derived Butantã strain (LT-BUT), MVA, or Wyeth (Dryvax) (Figure 1). The data confirmed that the viruses involved with the studied outbreaks are consistent with those involved in past described VACV zoonotic outbreaks in Brazil.

### 3.3. PBMCs from Infected Individuals Proliferated and Produced IFN*γ* after Stimulation with VACV Antigens

**Figure 2:**
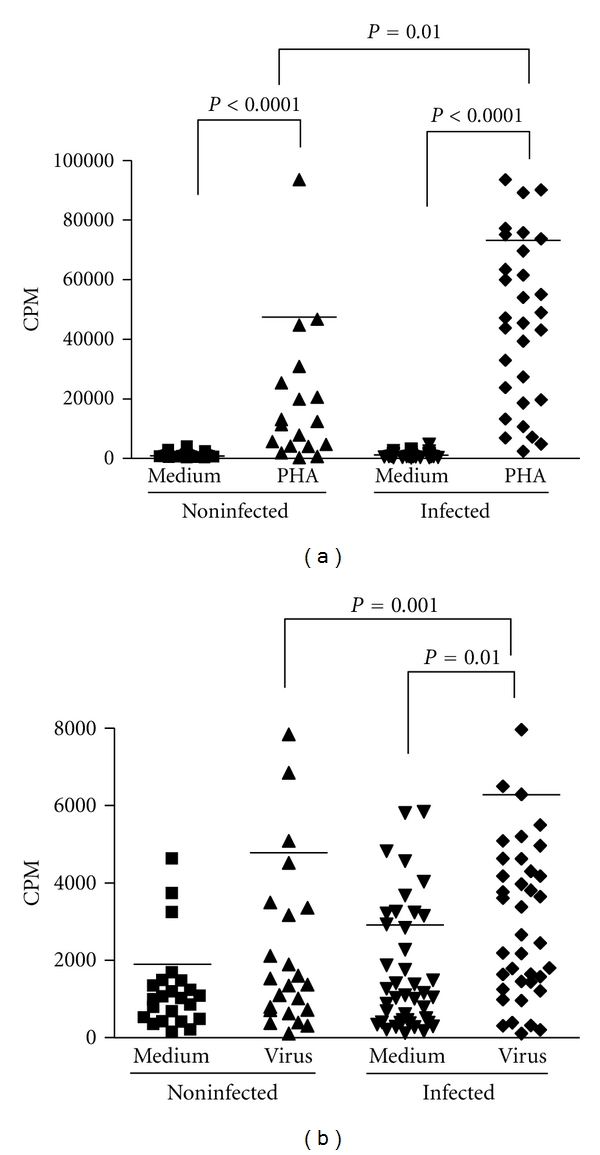
Proliferative responses in PBMCs of individuals infected or not by zoonotic *Vaccinia virus. *Peripheral blood mononuclear cells (PBMCs) from patients infected or not with zoonotic *Vaccinia virus *were cultured in the presence of PHA (a), UV-inactivated virus (b) or mock-treated (medium). After 6 days of stimulus, the cell proliferation was determined by [^3^H]thymidine incorporation. Statistical significance (*P* values), based on the median values of each group, is presented on the graphs. CPM: counts per minute.

**Figure 3:**
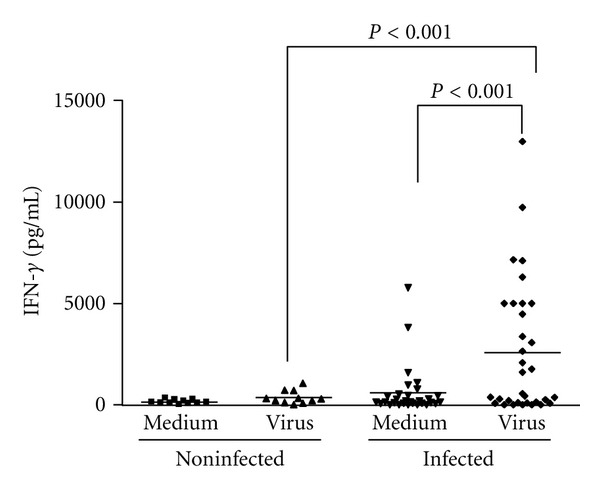
Interferon-gamma production in PBMCs of individuals infected or not by zoonotic *Vaccinia virus. *Peripheral blood mononuclear cells (PBMCs) from patients infected or not with zoonotic *Vaccinia virus *were cultured in the presence of UV-inactivated virus or mock-treated (medium). After 6 days of stimulus, the amount of IFN-*γ* produced in the cultures' supernatants was measured by cytometric bead array immunoassay. Statistical significance (*P* values), based on the median values of each group, is presented.

In order to evaluate the immune responsiveness of the infected individuals to VACV antigens, we stimulated their peripheral blood mononuclear cells (PBMCs) *ex vivo*. Cells were expose to either PHA (Figure 2(a)) or UV-treated VACV (Figure 2(b)), and cell proliferation was determined by [^3^H] thymidine incorporation. Upon mitogenic or antigenic stimulation, PBMCs from VACV-infected individuals presented a significantly (*P* = 0.01) higher cellular proliferative response when compared to PBMCs from noninfected subjects. Cells from both groups showed lower proliferative responses when mock-treated with culture medium, as expected (Figures 2(a) and 2(b)). Mathew and coworkers [35] demonstrated that PBMCs from individuals who received VACV immunization presented transient decreased proliferative responses to PHA, anti-CD3, and VACV antigens when comparing the proliferative responses from single individuals before and after vaccination. However, this was not observed when we compared antigen-induced proliferation in PBMCs from naturally infected patients to noninfected individuals. Next, we evaluated the levels of cytokines secreted by PBMCs on the culture supernatants of cells obtained from all subjects. The amounts of secreted IFN*γ* produced after VACV antigenic stimulation were significantly higher in infected individuals (*P* < 0.001) (Figure 3). This result differs from a previous observation in which a single VACV-infected patient was studied and presented diminished amounts of IFN*γ* in comparison to noninfected controls [8]. Mock-treated cells did not produce significant amounts of IFN*γ* (Figure 3). Analysis of IL-2, IL-4, IL-5, IL-10, and TNF-*α* from both groups did not show detectable amounts of these cytokines in culture supernatants. 

### 3.4. CD4^+^ T Cells, but Not CD8^+^ T Cells, Are Less Activated in Infected Patients When Compared to Noninfected Individuals

**Figure 4:**
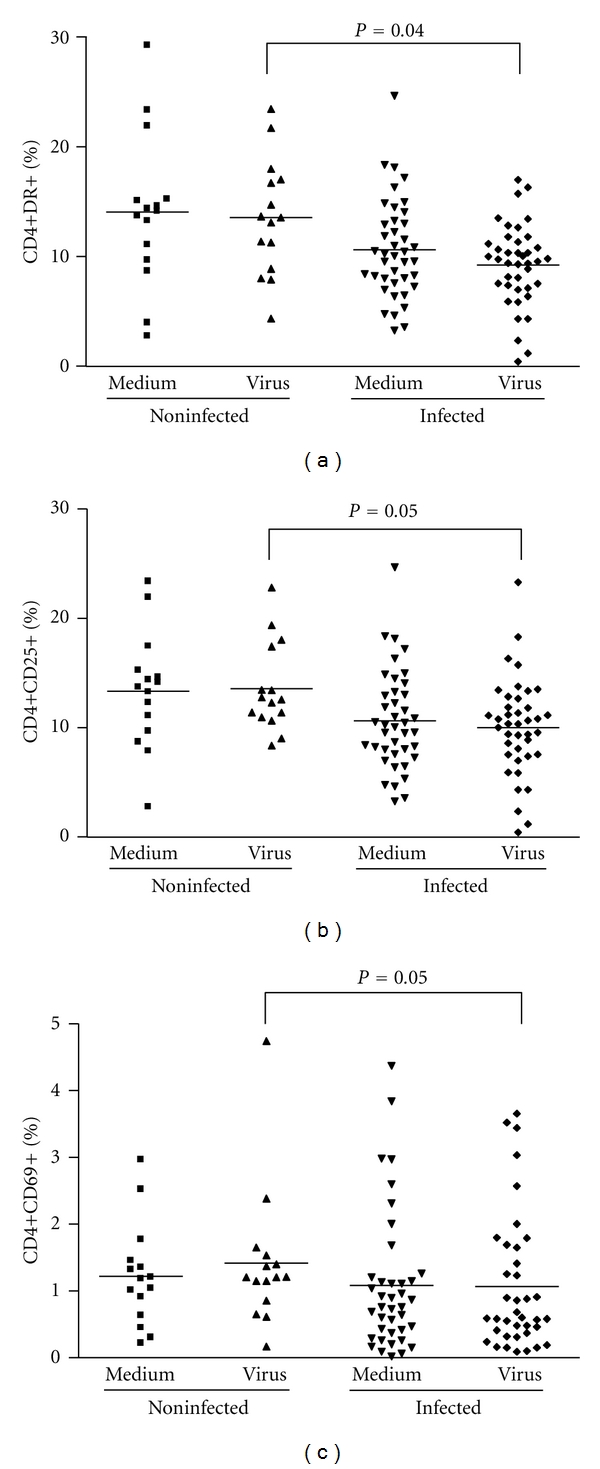
CD4^+^ T-cell activation status in PBMCs from individuals infected or not by zoonotic *Vaccinia virus. *Peripheral blood mononuclear cells (PBMCs) from patients infected or not with zoonotic *Vaccinia virus *were cultured in the presence of UV-inactivated virus or mock-treated (medium). After 72 hours of stimulus, cells were fixed, counted, and the following parameters were evaluated by flow cytometry using specific mouse anti-human antibodies: (a) percentage of CD4^+^ T lymphocytes expressing HLA-DR surface activation marker; (b) percentage of CD4^+^ T lymphocytes expressing CD25 surface activation marker; (c) percentage of CD4^+^ T lymphocytes expressing CD69 surface activation marker. Statistical significance (*P* values), based on the median values of each group, is presented on the graphs.

**Figure 5:**
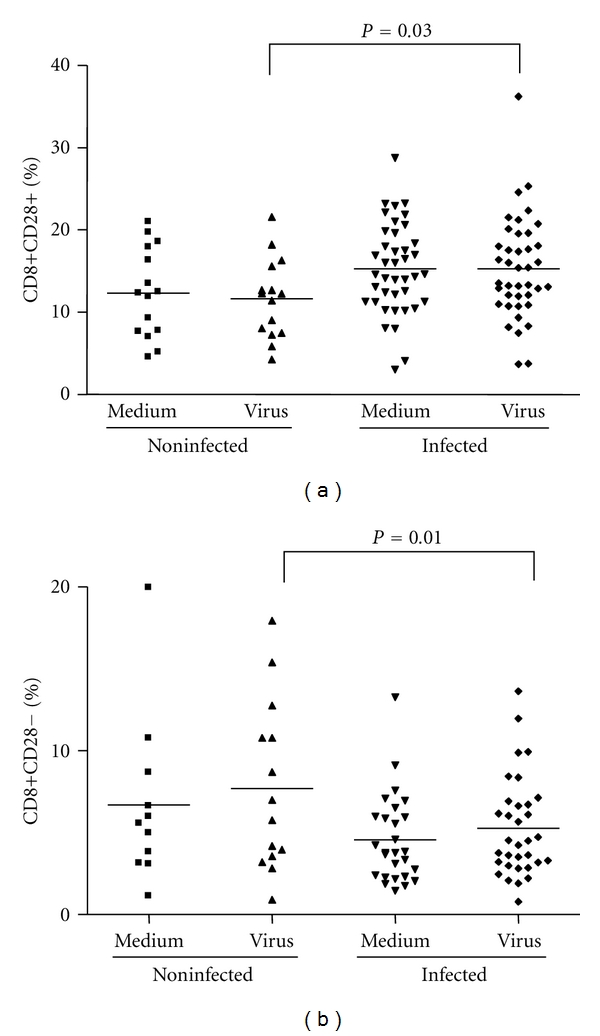
CD28 molecule expression and evaluation of regulatory T CD8^+^ cells in PBMCs from individuals infected or not by zoonotic *Vaccinia virus. *Peripheral blood mononuclear cells (PBMCs) from patients infected or not with zoonotic *Vaccinia virus *were cultured in the presence of UV-inactivated virus or mock-treated (medium). After 72 hours of stimulus, cells were labeled with mouse anti-human CD8 antibodies and anti-human CD28 antibodies. The percentages of CD8^+^ CD28^+^ (a) and CD8^+^ CD28^−^ (b) T-cell subsets were determined by flow citometry. Statistical significance (*P* values), based on the median values of each group, is presented on the graphs.

Analysis of the mean percentage of T and B lymphocytes, NK cells and monocytes were performed on PBMCs from both groups after *ex vivo *stimulation with UV-treated VACV. These analyses demonstrated an increase on the mean percentage of T cells (CD3^+^) (*P* = 0.008) and a surprisingly lower mean percentage of B lymphocytes (CD19^+^) (*P* = 0.03) in infected individuals (Table 1). Therefore, these results were associated with a significant increase in CD3^+^ : CD19^+^ ratio in infected individuals when compared to the noninfected group (*P* = 0.01). No significant differences were observed in the mean percentage of T lymphocyte subsets (CD4^+^ and CD8^+^) and in the mean values of monocytes (CD14^+^) and NK cells (CD16^+^) (Table 1). Apart from the unexpected decrease in total B cell amounts in infected patients, other results seemed to be consistent with typical late immune responses during an acute viral infection, especially considering T-cell responses. However, when T-cell subsets coexpressing CD25, CD69, CD28, CTLA-4, and HLA-DR, as activation markers, were analyzed on PBMCs from infected and noninfected individuals, a different picture emerged. Expression of HLA-DR, CD25, and CD69 on the surface of CD4^+^ T lymphocytes was lower in infected patients (*P* = 0.04, *P* = 0.05, and *P* = 0.05, resp.) (Figure 4). Importantly, we observed no differences in the cell activation status when PBMCs were either stimulated with VACV antigens or mock-treated. Expression levels of CD28 and CTLA-4 on CD4^+^ T lymphocytes from the two human groups were not significantly different (not shown). On the other hand, CD8^+^ T lymphocytes from infected individuals presented a significant increase in CD28 expression (*P* = 0.03) (Figure 5(a)). No significant differences were observed when cells were stimulated with VACV antigens or mock-treated. Analysis of HLA-DR, CD25, and CD69 on CD8^+^ T lymphocytes did not show any statistical differences between the groups (not shown). 

### 3.5. CD14^+^ Cells and B Cells Are Less Activated in Infected Patients When Compared to Noninfected Individuals. Relative Amounts of Regulatory CD8 T Cells Are Also Smaller in Infected Subjects

**Figure 6:**
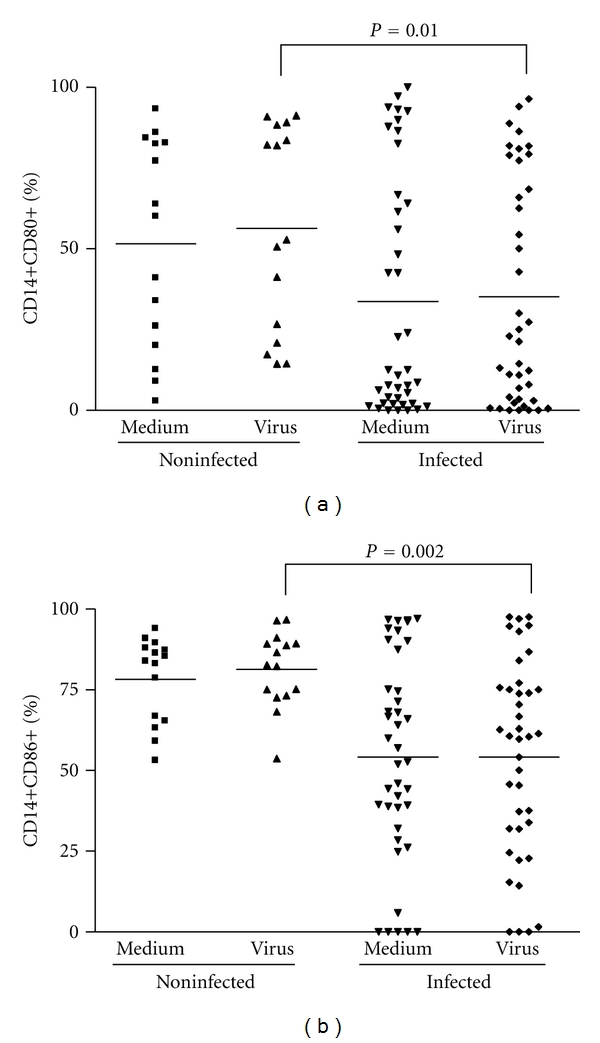
Monocyte (CD14^+^) activation status in PBMCs from individuals infected or not by zoonotic *Vaccinia virus. *Peripheral blood mononuclear cells (PBMCs) from patients infected or not with zoonotic *Vaccinia virus *were cultured in the presence of UV-inactivated virus or mock-treated (medium). After 72 hours of stimulus, cells were fixed, counted, and the following parameters were evaluated by flow cytometry using specific mouse anti-human antibodies: (a) percentage of monocytes (CD14^+^) expressing CD80 surface activation marker; (b) percentage of monocytes (CD14^+^) expressing CD86 surface activation marker. Statistical significance (*P* values), based on the median values of each group, is presented on the graphs.

**Figure 7:**
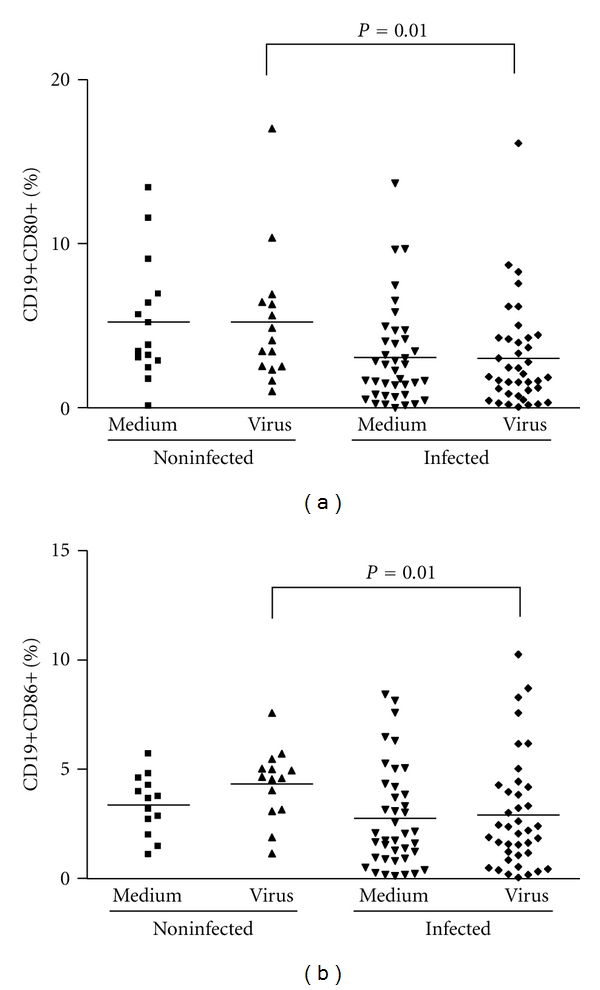
B cell (CD19^+^) activation status in PBMCs from individuals infected or not by zoonotic *Vaccinia virus. *Peripheral blood mononuclear cells (PBMCs) from patients infected or not with zoonotic *Vaccinia virus *were cultured in the presence of UV-inactivated virus or mock-treated (medium). After 72 hours of stimulus, cells were fixed, counted, and the following parameters were evaluated by flow cytometry using specific mouse anti-human antibodies: (a) percentage of B lymphocytes (CD19^+^) expressing CD86 surface activation marker; (b) percentage of B lymphocytes (CD19^+^) expressing CD80 surface activation marker. Statistical significance (*P* values), based on the median values of each group, is presented on the graphs.

Some Orthopoxviruses, such as the CPV and VACV, are known to interfere with the APC's functions by disrupting MHC classes I- and II-mediated antigen presentation [36–39]. Therefore, although monocytes were present in PBMCs from infected and noninfected patients in comparable amounts, it seemed appropriate to check whether these cells were activated. Thus, the expression of the activation markers CD80 and CD86 on the surface of macrophages/monocytes (CD14^+^) after stimulation with VACV antigens were evaluated. The percentage of monocytes expressing such markers was significantly lower in infected patients (*P* = 0.01 and *P* = 0.002, resp.) (Figures 6(a) and 6(b)). Likewise, the expression of CD80 and CD86 on the surface of B lymphocytes (CD19^+^) was measured. Not only the total amounts of B lymphocytes were lower in infected patients (Table 1), but these cells were also less activated when compared to uninfected individuals, as judged by the low percentage of CD19^+^ CD80^+^ and CD19^+^ CD86^+^ cells on PBMCs from the first group (*P* = 0.01) (Figures 7(a) and 7(b)). Finally, we also evaluated relative amounts of regulatory CD8^+^ T Lymphocytes (CD8^+^/CD28^+^ to CD8^+^/CD28^−^ ratio) in PBMCs from all subjects. PBMCs from infected subjects presented significant decrease (*P* = 0.01) in the relative amounts of regulatory CD8 T Lymphocytes when compared to noninfected individuals (Figure 5(b)). This is an interesting finding since increase in CD8^+^ regulatory cells have been demonstrated to inhibit CD4 proliferation. Overall, no significant differences were seen between cells stimulated with VACV antigens or mock-treated.

### 3.6. Mice Infected with a VACV Zoonotic Isolate Also Show Modulation in Specific Compartments of Their Immune Responses

**Figure 8:**
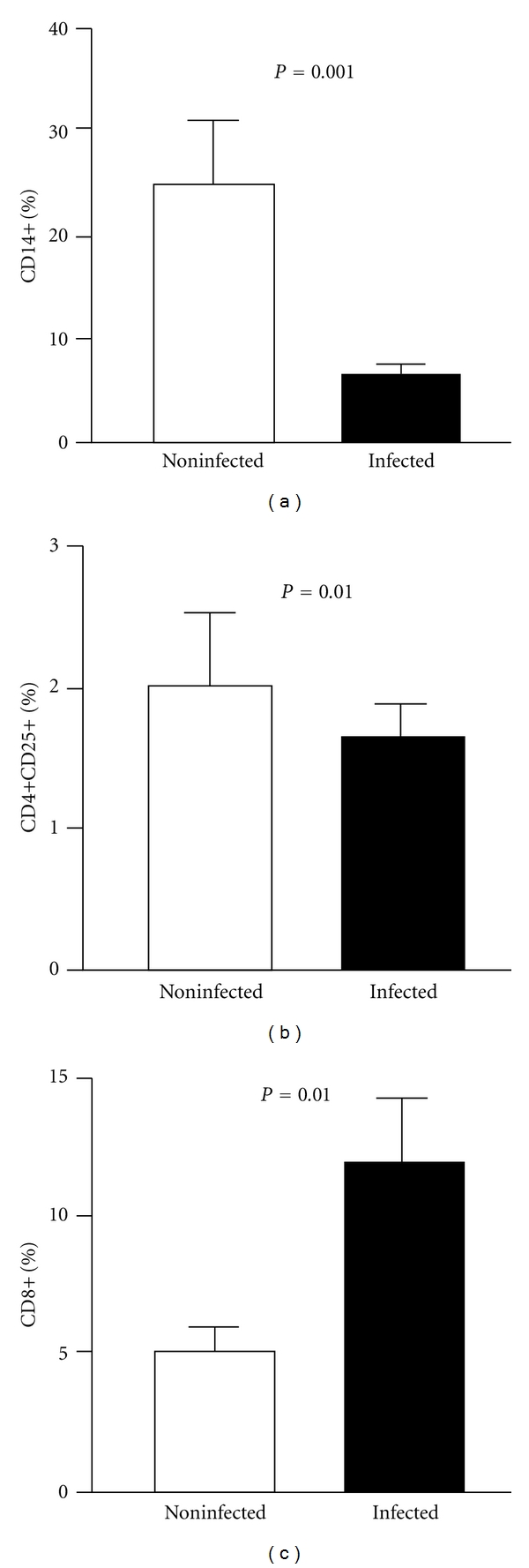
Aspects of the immune responses in mice infected or not with zoonotic *Vaccinia virus*. Peripheral blood mononuclear cells (PBMCs) from mice either infected with a zoonotic *Vaccinia virus *isolate or noninfected were cultured for 72 hours in the presence of UV-inactivated VACV. After incubation, cells were labeled with specific antibodies and analyzed by flow cytometry. (a) Percentage of total monocytes (CD14^+^); (b) percentage of CD4^+^ T lymphocytes expressing the CD25 surface activation marker; (c) percentage of CD8^+^ T lymphocytes. Bars represent the mean results from 10 animals. Error bars and *P* values are indicated.

In order to further confirm the zoonotic VACV's ability to modulate specific immune responses, we infected mice with an isolated VACV sample and analyzed the cellular immune response elicited during the onset of the acute disease. Although not all parameters studied for humans were evaluated in the mice experiments, the obtained results were comparable to those observed in infected persons. Total counts of macrophages/monocytes (CD14^+^ cells) were lower in the infected group (*P* < 0.001) (Figure 8(a)), and the expression of CD25 surface activation marker was diminished on CD4^+^ T cells when compared to noninfected animals (*P* = 0.01) (Figure 8(b)). Relative amounts of specific CD8^+^ T cells were higher in infected animals when compared to the noninfected group (*P* = 0.01) (Figure 8(c)).

## 4. Discussion

*Orthopoxvirus*-mediated modulation of immune responses in humans is poorly demonstrated due to the inherent difficulties to study populations infected by virulent virus strains. In this respect, Hammarlund and colleagues [19] have shown that MPV infecting human cells is able to evade CD4^+^ and CD8^+^ T cell responses through an MHC-independent mechanism, although, in this case, they did not evaluate immune responses in acutely infected patients.

On a previous work we had shown that one studied VACV-infected patient produced low amounts of IFN*γ* [8]. However, when a larger group of infected patients were analyzed in the current study, this pattern was not observed. Variations on the levels of IFN*γ* produced by different individuals is usually high, as exemplified by the individual dot distribution in Figure 3, and that may explain the fact that this cytokine level was low on the study performed before. Indeed, cytokine production may vary enormously in field patients as a result of different ages; different genetic backgrounds; other existing microbial infections; immunological and nutritional status; general health conditions. Such variations in IFN*γ* production has been seen in field studies of malaria, for instance, among other infectious diseases [40, 41].

We have also shown that specific compartments of the human immune response to zoonotic VACV acute infections are virus-modulated. That could be inferred from a marked virus-induced decrease in the activation status of CD4^+^ T cells, B cells, and macrophage/monocytes. This apparent bias in the virus immune evasion strategy may suggest that these specific responses are those responsible for the impairment of VACV replication success in the human host. Indeed, viruses in general, and specially poxviruses (due to their large coding capacity), encode a multitude of proteins that interfere with diverse immunological functions of the host [42–44]. Moreover, viruses are usually very didactical in demonstrating which components of the host immune system are determinants to their replicative success by encoding evasion proteins that specifically affect such components. The fact that humans acutely infected by virulent VACV showed an apparent virus-induced modulation of macrophages/monocytes, CD4^+^ T and B cells suggests that these may be essential and perhaps sufficient for virus clearing and disease resolution during an acute, primary infection. Indeed, VACV disruption of MHC class-II-restricted antigen presentation has been described [37–39]. On the other hand, we found that, CD8^+^ T cell responses are apparently not modulated by the infections, suggesting that CD8^+^ T cell responses may have a lesser impact on the resolution of VACV primary infections, as hypothesized by some authors [20, 28–31]. One limitation to the study is the size of the analyzed group, which comprises 53 orthopoxvirus-infected human patients and 18 noninfected individuals living in the same affected areas. Notification of human poxvirus infections is not mandatory in Brazil, and identification of patients is done through active random search. The fact has impaired our ability to enroll a larger number of patients. At this point, we cannot provide mechanistic explanations for the findings presented here. To that end, mice infections with mutant viruses lacking specific immune evasion genes could help to understand how orthopoxviruses induce such specific and polarized modulation of the host immune response.

The VACV outbreaks taking place in Brazil represent an important opportunity to understand aspects of naturally acquired *Orthopoxvirus *infections in a population scale. Although of undeniable importance, most studies on human immune responses to primary *Orthopoxvirus *infections have been carried out in voluntary recipients of the smallpox vaccine. Because *Orthopoxvirus*' infections in humans are relatively rare nowadays, vaccinated persons represent the most obvious alternative to study immune responses to these viruses in a population scale. However some aspects must be considered. First, vaccine strains are usually chosen because they are attenuated and avirulent, and only cause adverse events on a small number of individuals under very specific conditions [45, 46]. Thus, the responses elicited against them may not be necessarily identical to the immune responses generated during infection with a more virulent *Orthopoxvirus *strain. Secondly, the attenuation process frequently leads to the loss of genes that are not essential for virus growth *in vitro*, such as those involved in host's immune evasion mechanisms. Therefore, VACV vaccine strains generally lack many such genes. This is the case of the Dryvax strain, which lacks genes coding for the interferon (IFN)-*α*/*β* viroceptor, an ankyrin-like protein (ortholog of VARV-BSHB18R) and tumor necrosis factor (TNF)-*α* receptor homolog [47]; and the Modified Vaccinia Ankara (MVA) strain, which lacks many immune-evasion genes and is not replicative in most mammal cells [48]. These viruses' relative deficiency in modulating host's immunity may result in infections whose patterns are definitely different from those caused by more virulent *Orthopoxvirus *strains. Finally, most studies are carried out in controlled environments and conditions which include predetermined human populations; inoculum size; sites of inoculation; careful followup of vaccinees. These conditions, although necessary, do not mimic natural infections, and that may have an impact on how the individual's immune system responds to the infection.

## 5. Conclusions

Despite the fact that immunomodulatory genes are present in the genome of VARV, the most notorious human poxviral pathogen [49], the impact of virus-encoded immune evasion strategies during human infections by poxviruses is largely unknown. We have studied 53 patients acutely infected by zoonotic VACVs and analyzed their innate and adaptative immune responses. Our results suggested that specific cell subsets including B cells, CD4^+^ T cells, and macrophage/monocytes are specifically modulated by the infection, in comparison to uninfected patients. On the other hand, CD8^+^ T cell responses seem to be unaltered, mirroring typical CTL response patterns during a viral infection. These results are compatible with a model in which CD4^+^ T cell-dependent antibody responses are the main responsible for disease control and virus clearance during primary Orthopoxvirus' infections [28, 30]. This study represents the first attempt to analyze aspects of the human immune responses during the onset of acute, naturally acquired infections by orthopoxviruses.

## Figures and Tables

**Table 1:** Mean percentage of T (CD3^+^, CD4^+^, CD8^+^) and B (CD19^+^) lymphocytes, NK cells (CD16^+^) and monocytes (CD14^+^) on PBMCs from noninfected or zoonotic *Vaccinia virus* infected individuals after stimulation with virus antigens.

	Cell phenotype					
Groups	CD3^+^	CD4^+^	CD8^+^	CD19^+^	CD16^+^	CD14^+^
Noninfected	72.5 ± 3.9	52.1 ± 5.2	19.2 ± 2.1	16.4 ± 3.3	22.8 ± 6.5	2.9 ± 1.4
Infected	79.2 ± 4.9*	58.3 ± 3.2	20.3 ± 2.0	11.4 ± 2.5**	19.8 ± 7.4	4.4 ± 1.4

PBMCs: peripheral blood mononuclear cells.

*P = 0.008, when infected and noninfected groups were compared.

**P = 0.03, when infected and noninfected groups were compared.
